# Metamemory and aging: Psychometric properties of the Brazilian version of the Multifactorial Memory Questionnaire for elderly

**DOI:** 10.1590/S1980-5764-2016DN1002007

**Published:** 2016

**Authors:** Sharon Sanz Simon, Renata Thomas Ávila, Gilson Vieira, Cássio Machado de Campos Bottino

**Affiliations:** 1 Old Age Research Group (PROTER), Department of Psychiatry, Faculty of Medicine, University of São Paulo, Brazil.; 2 Inter-institutional Grad Program on Bioinformatics, University of São Paulo, Brazil.

**Keywords:** metamemory, subjective memory, episodic memory, cognition and aging

## Abstract

Metamemory measures provide subjective memory information and are relevant to investigate memory ability in aging. However, there is a lack of metamemory instruments available in Brazil.

**Objective:**

The aim of this study was to examine the psychometric properties of the Brazilian version of the Multifactorial Memory Questionnaire (MMQ), which evaluates different dimensions of subjective memory functioning, such as Feelings, Abilities and Strategies used in everyday life.

**Methods:**

The MMQ was translated into Portuguese and administered to 30 Brazilian elderly subjects. The participants underwent cognitive tests, mood scales and metamemory instruments.

**Results:**

Analyses revealed good internal consistency (Cronbach's a coefficient ranged from 0.75 to 0.89) and test-retest validity for each MMQ dimensions (positive correlations between two applications ranged from 0.75 to 0.8). Convergent validity evidence for the MMQ was confirmed by significant positive correlations (0.47 to 0.68) with dimensions of the Metamemory in Adulthood scale (MIA) (i.e., the Ability, Control, Self-efficacy and Strategy dimensions). Discriminant validity revealed no associations between the MMQ and cognitive performance, suggesting a weak metamemory-objective memory correspondence. Moreover, there was a negative correlation between MMQ-Ability subscale scores and mood symptoms (-0.63 for anxious symptoms, and -0.54 for depressive symptoms); and the Brazilian MMQ was comparable with MMQ translations to other languages.

**Conclusion:**

The Brazilian MMQ exhibits good psychometric properties and appears promising for clinical and research purposes. Additional studies are needed to further examine the psychometric properties of the Brazilian MMQ in a larger sample.

## RESUMO

Medidas de metamemória fornecem informações acerca da memória subjetiva e são relevantes para se compreender a habilidade de memória no envelhecimento. Todavia, há uma falta de instrumentos de metamemória disponíveis no Brasil.

**Objetivo:**

O presente trabalho teve como objetivo examinar as propriedades psicométricas da versão brasileira do Questionário Multifatorial de Memória (MMQ), que avalia diferentes aspectos da memória subjetiva, como Sentimentos, Habilidade e Estratégias usadas na vida diária.

**Métodos:**

O MMQ foi traduzido para o Português e administrado em 30 idosos brasileiros. Os participantes realizaram testes cognitivos, escalas de humor e questionários de metamemória.

**Resultados:**

Análises revelaram boa consistência interna (coeficiente a de Cronbach's variou de 0.75 a 0.89) e validade teste-reteste para cada dimensão do MMQ (correlações positivas entre as duas aplicações variou de 0.75 a 0.8). Validade convergente do MMQ foi identificada através de correlações positivas (0.47 a 0.68) com as dimensões do *Metamemory In Adulthood Questionnaire* (MIA) (i.e., Habilidade, Controle, Autoeficácia e Estratégia). Validade discriminante revelou ausência de associação entre MMQ e performance cognitiva, sugerindo uma fraca correspondência entre metamemória e memória objetiva. Além disto, observou-se uma correlação negativa entre os escores da subscala MMQ-Habilidade e sintomas do humor (-0.63 para sintomas ansiosos, e -0.54 para sintomas depressivos); e o MMQ Brasileiro se mostrou comparável a traduções do MMQ em outros idiomas.

**Conclusão:**

O MMQ Brasileiro apresenta boas propriedades psicométricas e parece ser promissor para o uso clínico e de pesquisa. Estudos adicionais são necessários para investigar caracteristicas psicométricas do MMQ Brasileiro em uma amostra maior.

## INTRODUCTION

Metamemory is frequently defined as knowledge about one's own memory functioning,^1^ ^-^ ^3^ and the monitoring and control processes that allow subjects to regulate their memory activity and content.^4^ Knowledge about one's own memory includes factual knowledge about tasks, memory strategies, and the subject's beliefs about their memory abilities.^5^ These aspects are considered important in directing the use of memory processes in overall decision making.^6^ Also, authors have argued that the metamemory definition should also include feelings and emotions about memory, along with self-efficacy associated with memory,^5^ ^,^ ^7^ which is related to confidence level regarding one's own memory ability.^8^ Metamemory has been studied within the field of psychology of aging to understand changes in memory abilities during aging. Also, in older adults, beliefs in memory efficacy appear to be weaker than in younger individuals,^7^ ^,^ ^9^ where elders tend to be convinced of a decline over time and report less control over their memory function than younger adults.^10^ ^,^ ^11^ Other evidence suggests that beliefs about one's potential to use memory effectively influence the self-selected exposure to memory-demanding situations, degree of effort and actual performance.^9^ ^,^ ^12^ ^-^ ^14^

Metamemory research has been considered relevant since subjective memory complaints (SMCs) are frequent in elders, and can be associated with a high level of distress and reduced quality of life, although do not necessarily interfere with activities of daily living.^15^ Also, the occurrence of SMCs is one of the DSM-5 diagnostic criteria for Mild Neurocognitive Disorder, together with objective cognitive deficits identified by means of neuropsychological batteries.^16^ Moreover, the use of memory strategies play an important role in the ability of older adults to adapt to or compensate for late-life memory loss and impairment,^17^ with recent findings suggesting that elders preferentially use external memory strategies to cope with everyday memory impairment due to aging.^18^

In recent decades, self-report memory questionnaires have been developed to better investigate metamemory. These instruments are useful tools for investigating everyday memory problems, estimating the clinical significance of a memory problem and in guiding the development and evaluation of intervention programs.^19^ Also, they can provide important information about aspects of subjective performance that is not captured by objective memory testing alone. However, studies describe weak metamemory-objective memory correspondence, suggesting that other factors can contribute to metamemory, such as mood (e.g. depressive and anxiety symptoms), and executive dysfunction.^5^ ^,^ ^20^ These aspects can lead individuals to inaccurately rate their memory abilities. It is noteworthy that, in general, these instruments provide information primarily or exclusively about the frequency that memory mistakes occurs whereas few provide information regarding the individuals's knowledge, beliefs and feelings about their memory or on the strategies used in everyday life.^17^ The Memory Function Questionnaire (MFQ)^21^ and the Metamemory in Adulthood Questionnaire (MIA)^22^ overcome these shortcomings.

Despite the considerable number of self-report memory questionnaires described in the literature, Troyer and Rich^19^ list some drawbacks associated with their use, especially in clinical settings. First, some instruments were developed primarily for research, so the items do not necessarily reflect aspects of memory that are amenable to clinical intervention. Second, several instruments include items that are not applicable to some individuals, such as public speaking, reading novels, driving or working. Third, some questionnaires, such as the MIA, contain a very large number of items and are time consuming, which can affect compliance, especially in older or cognitively impaired individuals.

Therefore, Troyer and Rich^19^ designed the Multifactorial Memory Questionnaire (MMQ), a metamemory questionnaire developed for application in clinical and research settings. The MMQ consists of three parts designed to assess dimensions of metamemory judgments: (1) contentment with one's own memory ability (MMQ-Contentment); (2) perception of everyday memory ability (MMQ-Ability); and (3) everyday memory strategies (MMQ-Strategy). To increase compliance, the authors developed a shorter questionnaire than previously available instruments, requiring about 10 minutes to complete. Thus, the MMQ can be considered an improvement over other memory self-report instruments because it embodies a number of features in combination: multidimensionality, clinical relevance, brevity and ease of administration. Moreover, the questionnaire includes items pertaining to aspects of memory-related affects, not typically included in existing questionnaires. 

Regarding the psychometric properties of the MMQ, it has been confirmed as having excellent content validity, factorial validity, reliability (test-retest and intratest), construct validity (convergent and discriminant) and reliability of independent demographic variables.^17^ More specifically, high internal consistency in each dimension was found (Cronbach's a was 0.95 for MMQ-Contentment, 0.93 for MMQ-Ability, and 0.83 for MMQ-Strategy).^19^ Also, the convergent validity of the MMQ scales was demonstrated by their correlations with the MFQ and MIA, and discriminant validity was demonstrated by the lack of a correlation between the MMQ scales and cognitive tests. Therefore, the MMQ exhibits psychometric strengths and has been translated to other languages.^24^ ^-^ ^26^ Furthermore, the MMQ has been described as a useful measure for several population samples, such as patients with Mild Cognitive Impairment (MCI) and epilepsy.^27^ ^,^ ^28^

The majority of the metamemory studies and questionnaires cited above were performed in elderly populations of developed countries with different cultural backgrounds to Brazil. Thus, it is crucial to verify whether results in the international literature are also valid for the Brazilian population. For instance, whether there is a similarly weak metamemory-objective memory correspondence; or an impact of mood, education and/or cultural background on metamemory measures. Hence, to better develop this research field in the country, Brazilian studies need to translate and validate metamemory instruments. Although some efforts have been made such as with the Brazilian version of the MIA,^7^ there is still a lack of metamemory questionnaires available in Brazil. Also, since the MMQ has been considered a useful tool in different countries,^19^ ^,^ ^24^ ^-^ ^26^ we believe that developing a Brazilian version of the MMQ can be a relevant contribution to our population. 

Therefore, the aim of the present study was to develop a Brazilian version of the MMQ and to examine its psychometric properties as a tool for research and clinical purposes.

## METHODS

Translating and adapting the MMQ. The MMQ was translated in three stages. First, two clinical neuropsychologists trained in the aging field produced an independent translation from English to Portuguese. Second, these versions were unified to reach a consensus on the Portuguese version. Finally, a professional Portuguese-English translator back-translated the material. The final version is given in the Appendix. 

Participants. The study participants were 30 cognitively normal elders aged 60 years or older. The sample was recruited via community lectures, associations, a database of research volunteers and through recommendation by colleagues or volunteers. Participants were informed of the voluntary and anonymous nature of the research, and the study was approved by the Ethics Committee of the Medical School of the University of São Paulo.

Screening and cognitive evaluation. In order to identify participants with possible memory impairment, neuropsychologists initially conducted an individual interview and cognitive screening. Volunteers were excluded if they presented a history of psychiatric, neurological and/or medical condition that could affect cognition, as well as significant mood symptoms (9 or more points on the Hospital Anxiety and Depression Scale - HADS^29^) or inconsistent cognitive performance for age and educational level on the Mini-Mental State Examination (MMSE)^30^ ^,^ ^31^ or on measures of attention, working memory, learning and memory: Digit Span - forward and backward (WAIS-III),^32^ Rey Auditory Verbal Learning Test (RAVLT)^33^ and Brief Visual Memory Test - Revised (BVMT-R).^34^

Metamemory questionnaires. The participants filled out two metamemory questionnaires, the MMQ and the MIA. Although these are self-report questionnaires, the interviewer assessed possible difficulties in understanding the items. As briefly outlined earlier, the MMQ consists of 57 items, divided into three scales related to dimensions of metamemory judgments (Table 1): MMQ-Contentment, MMQ-Ability; MMQ-Strategy. The subjects were asked to evaluate these dimensions based on the last 2 weeks, as required by the MMQ instructions.^19^ The Portuguese forms of the MMQ are provided in the supplementary material.

**Table 1:** MMQ questionnaire dimensions.

Dimensions	Content	Format	Interpretation
MMQ-Contentment	Feelings about own memory ability	18 items on 5-point Likert scale	Higher scores = greater contentment
MMQ-Ability	Frequency of forgetting in different situations	20 items on 5-point Likert scale	Higher scores = fewer memory problems; better ability
MMQ-Strategy	Mnemonic Strategies use in everyday life	19 items on 5-point Likert scale	Higher scores = greater use of strategies

MMQ-Contentment addresses several emotions and perceptions that subjects may have about their current memory ability. Statements address positive emotions (e.g. confidence, satisfaction), negative emotions (e.g. embarrassment, irritation), and subjective ability ratings (e.g. comparison to peers, belief that one has a serious memory problem). Respondents rate their level of agreement with each statement according to how they felt in the past 2 weeks. MMQ-Ability contains everyday memory situations, such as remembering appointments, paying bills, and names. Responders indicated the frequency with which each mistake occurred over the last 2 weeks. MMQ-Strategy describes different memory aids and strategies applicable to everyday memory tasks, such as writing appointments on a calendar or agenda, using visual imagery, repeating information to oneself. Responders indicated the frequency with which each strategy was used over the last 2 weeks. 

Regarding the MIA,^22^ the questionnaire comprises 108 items, assessing the following dimensions: Strategy (memory strategy use or knowledge about strategies), Task (knowledge about memory processes), Ability (perceptions about one's own ability to memorize), Change (perceived evolution of one's memory with aging), Activity (activities maintaining memory), Anxiety (stress related to memory situations), Motivation (importance of succeeding in memory tasks), and Locus (locus of control in memory abilities). Also, two factors can be extracted from the grouping of some items: Self-Efficacy factor, result of the sum of Ability, Locus and Change items; and Self-Knowledge factor, result of the sum of Strategy, Task and Motivation items. 

Procedures. Questionnaires, cognitive tests and the mood scale were administered individually and the MMQ were applied in a fixed order (Contentment, Ability and Strategy). MMQ and cognitive test scores were compared to investigate the *discriminant validity* of the MMQ, and to better understand metamemory-objective memory correspondence. Also, *convergent validity* was investigated by comparing the performance between MMQ and MIA, since MIA has already been validated for the Brazilian population.^7^ Three months later, the participants were resubmitted to MMQ to assess the *test-retest reliability* of the instrument. Furthermore, MMQ scores were compared with the scores of other populations.

Statistical analysis. Internal consistency of each MMQ dimension was evaluated using Cronbach's a coefficient for which a value of 0.70 was considered acceptable.^23^ Test-retest reliability of the Brazilian version of the MMQ was assessed by calculating Pearson's product moment correlation coefficient between the first and second measurement. Convergent validity between the MMQ and MIA scores was investigated using Pearson's product moment correlation coefficient with results corrected for multiple comparisons (Bonferroni method). For these two analyses, the average between the first and second measurement of the MMQ scores was considered. Moreover, discriminant validity was assessed using linear regression models to evaluate the association between the MMQ scores and cognitive test performances. Finally, Welch's *t*-test was used to compare the MMQ mean scores with scores of different populations.

## RESULTS

Sample. Thirty cognitive normal elders were included in the study. Participants' mean age was 75.2 years (ranging from 60 to 96, SD = 10.8), and mean education in years was 12.4 years (ranging from 4 to 22, SD = 3.8). Moreover, 80% of the participants were female, 60.3% were professionally active, 76.6% lived in their own homes and 23.3% lived in a retirement home. All participants performed within the age and education appropriate norms of the objective cognitive tests applied, as shown in Table 2.

**Table 2:** Participants' characteristics and cognitive performance.

Measures	Mean ± SD
Age	75.2 ± 10.8
Years of education	12.4 ± 3.8
MMSE	28.6 ± 1.5
Digit Span	14.6 ± 3.1
RAVLT Immediate*	41.7 ± 10.2
RAVLT Delayed	8.3 ± 2.7
BVMT-R Immediate**	18.4 ± 8.0
BVMT-R Delayed	7.8 ± 3.3
HADS Anxiety	3.27 ± 2.75
HADS Depression	2.70 ± 2.39

BVMT-R: Brief Visuospatial Memory Test Revised; HADS: Hospital. Anxiety and Depression Scale; MMSE: Mini-Mental State Examination; RAVLT; Rey Auditory Verbal Learning Test; SD: Standard Deviation. *Sum of 5 trials; **Sum of three trials.

Internal consistency. The internal consistencies of the scores on the items constituting each MMQ dimension were examined using Cronbach's a coefficients on data from all participants. These analyses indicated highly reliable scores on all dimensions: for the Contentment dimension, a was 0.89 (0.95 in the original version^19^); for Ability, a was 0.87 (0.93 in the original version); and for Strategy, a was 0.75 (0.83 in the original version).

Test-retest reliability. Three-month test-retest reliability was examined and a high level of agreement was observed between the two applications of the Brazilian version of the MMQ. Significant correlations indicated highly reliable scores on all MMQ dimensions, suggesting satisfactory temporal stability of the Brazilian version of the MMQ (Table 3). It is noteworthy that the average time to complete the MMQ was around 10 to 15 minutes.

**Table 3:** Test-retest reliability: correlations between MMQ applications.

	Correlation*	P-value
MMQ Contentment	0.80	<0.001*
MMQ Ability	0.74	<0.001*
MMQ Strategy	0.75	<0.001*

MMQ: Multifactorial Memory Questionnaire; *Pearson's product moment correlation coefficient.

Convergent validity. As described in previous studies, convergent validity between the MMQ and the MIA was determined by calculating correlation coefficients,^19^ ^,^ ^25^ and adjusted for multiple comparisons (Table 4). 

**Table 4:** Comparisons between MMQ, MIA and HADS scores.

	MMQ Contentment	MMQ Ability	MMQ Strategy
MIA Ability	0.49*	0.57**	
MIA Control		0.47*	
MIA Self-efficacy	0.47*	0.58**	
MIA Strategy			0.68**
HADS Depressive symptoms		-0.54*	
HADS Anxious symptoms		-0.63**	

HADS: Hospital Anxiety and Depression Scale; MIA: Metamemory in Adulthood Questionnaire; MMQ: Multifactorial Memory Questionnaire; Significant correlations: Pearson's product moment correlation coefficient. **p ≤ 0.01; *p ≤ 0.05.

The Contentment dimension of the MMQ was significantly correlated with the Ability item and Self-Efficacy factor (sum of Ability, Locus and Change items) on the MIA (r = 0.49, p < 0.05; and r = 0.47, p < 0.05). These results indicate that a high degree of memory satisfaction was significantly associated with more positive perceptions about one's own ability to memorize (Ability), and possibly better locus of control in memory abilities and perceptions of memory changes related to the aging process (Locus and Change items).

The MMQ Ability scale showed significant correlations with MIA Ability (r = 0.47, p < 0.05), as reported in the literature,^19^ ^,^ ^25^ and also for the MIA Locus (r = 0.47, p < 0.05) and Self-efficacy factor (r = 0.58, p < 0.01). This association indicates that an optimistic rating of one's abilities was associated with few reports of difficulty,^25^ and better perceived self-efficacy in everyday life. Interestingly, a significant negative correlation between memory ability self-appraisal (MMQ-Ability) and mood symptoms was also observed, with stronger correlation with anxious symptoms (r = -0.63, p < 0.01) than depressive symptoms (r = -0.54, p < 0.05).

The MMQ Strategy dimension was strongly and significantly correlated with the Strategy dimension of the MIA, as reported by other authors^19^ ^,^ ^25^ (r = 0.68, p < 0.01).

Discriminant validity. In the original study of Troyer and Rich,^19^ a lack of correlation was shown between the MMQ scales and cognitive tests. Similarly, scores on the Brazilian version of the MMQ showed no correlation with any of the cognitive tests, suggesting weak metamemory-objective memory correspondence.

Comparisons between different samples and languages: The Brazilian MMQ scores are shown in Table 5, and scores for other samples, such as English-speaking (original sample),^19^ Spanish-speaking,^24^ French-speaking,^25^ are given in Table 6. When comparing the Brazilian sample with the original sample, the results show that the Portuguese-speaking sample had a similar pattern on the Contentment dimension, albeit somewhat lower (t = 1.7814; df = 143; p-value = 0.07), as well as higher Ability scores (t = 5.9293; df = 143; p-value < 0.001) and lower scores on the Strategy dimension (t = 9.0862; df = 143; p-value < 0.001). However, Brazilian scores showed a pattern more similar to those observed in the French and Spanish versions (p-values >0.05). 

**Table 5:** Scores obtained on Brazilian version of MMQ scales.

Measure	Contentment (18 Items) N = 30	Ability (20 Items) N = 30	Strategy (19 Items) N = 30
Mean	44.0	58.3	21.8
SD	12.4	9.4	9.1
Range	12-66	44-78	4-39

N: number; SD: Standard Deviation.

**Table 6:** Scores obtained on MMQ scales in English (original), French and Spanish.

	Cont Contentment				Ability				Strategy		
	English* (N = 115)	French** (N = 294)	Spanish^#^ (N = 67)		English* (N = 115)	French** (N = 294)	Spanish^#^ (N = 67)		English* (N = 115)	French** (N = 294)	Spanish^#^ (N = 67)
Mean	39.0	45.0	45.9		45.0	53.9	55.3		40.1	26.8	23.3
SD	14.0	10.5	11.4		11.3	9.2	12.4		10.0	10.2	13.1
Range	9-72	-	-		0-77	-	-		9-74	-	-

N: number; SD: Standard Deviation. *English-speaking sample: Mean age: 71.7 / Mean education (years): 13.8; **French-speaking sample: Mean age: 65.9 / Mean education (years): not provided; 33.9 % had 12 or more years of education; ^#^Spanish-speaking sample: Mean age: Not provided, range 61-81years / Mean education (years): not provided.

## DISCUSSION

The aim of the present study was to adapt the MMQ^19^ to the Portuguese language for use in the Brazilian population. Our results showed that the Brazilian MMQ had psychometric strengths for use in older adults. We observed good internal consistency (Cronbach's a coefficients ranged from 0.75 to 0.89) and test-retest reliability on each of the MMQ dimensions, along with temporal stability between the two applications (correlation coefficients ranged from 0.74 to 0.8). Discriminant validity was evidenced by the lack of correlation between memory self-appraisals and cognitive performance, and convergent validity by the positive correlations (from 0.47 to 0.68) between dimensions on the MMQ and MIA (i.e., Ability, Control, Self-efficacy and Strategy), akin to preview studies.^19^ ^,^ ^25^

 The comparisons between different MMQ languages showed that our Brazilian Portuguese-speaking sample had similar scores on the Contentment dimension, higher scores on the Ability dimension and lower scores on the Strategy dimension than the English-speaking sample of the original MMQ study.^19^ However, Brazilian scores showed a pattern similar to those observed in the Spanish-speaking^24^ and French-speaking^25^ samples. These differences may be explained in part due to translational aspects and cultural bias, which can influence perception about memory and aging, the small Brazilian sample size, and/or demographic differences between the samples, such as age and education. For instance, the Brazilian sample was slightly older (mean age: 75.2; SD: 10.8) than the original sample (mean age: 71.1; SD: 9.9) and slightly lower mean education (Brazilian sample mean age: 12.4; SD: 3.8; English-speaking sample mean age: 13.8; SD: 2.8). The exact influence of these factors on the MMQ scores remains unclear. However, it has been suggested that educational level can influence metamemory, with evidence that older adults with higher educational level have a more accurate level of confidence in retrospective metamemory.^6^ It is worth noting that in our study the MMQ was suitable for elders with different educational background (4 to 22 years of schooling). However, we recommend that examiners supervise individuals with low educational level more closely in order to check for instruction comprehension and possible mistakes.

 Similar to previous studies,^5^ ^,^ ^20^ a weak metamemory-objective memory correspondence was observed by the lack of relationship between memory self-appraisals and cognitive performance (discriminative validity). However, a significant negative correlation was found between self-appraisal memory abilities (MMQ-Ability) and mood symptoms (-0.63 for anxious symptoms, and -0.54 for depressive symptoms), suggesting that elders with less mood symptoms report better memory ability. These results are in tune with the literature suggesting that the relationship between metamemory and objective memory performance is probably mediated by other variables, such as mood symptoms,^35^ ^,^ ^36^ executive dysfunction,^20^ and also demographic characteristics, such age and educational background.^37^

Furthermore, future studies should investigate the psychometric properties of the external and internal strategies present in the MMQ Strategy separately,^25^ ^,^ ^26^ which can be a useful tool for evaluating the use of these different kinds of strategies and planning cognitive interventions. For instance, Fort et al.^25^ found a correlation between the MMQ Strategy dimension (external strategies) and both age and education.

The present study has some limitations that should be considered. First, the present sample size is small; and second, we did not present a measure of general health, which could be helpful to better characterize the sample, data which we collected only in a qualitative way through an interview. 

To our knowledge, there is a lack of metamemory instruments available in Brazil and the current study presents exploratory data regarding the Brazilian version of the MMQ, considered a promising self-report measure of memory, given its focus on clinically relevant aspects of memory, multidimensionality, brevity, and ease of administration.^19^ ^,^ ^24^ ^-^ ^26^ The MMQ has been considered sensitive to changes after cognitive intervention (e.g.. cognitive training and rehabilitation) in healthy older adults and MCI patients, having proven a useful tool for future studies.^27^ Also, the MMQ can be relevant to help design cognitive interventions in older adults who have cognitive complaints but no memory strategies in everyday life. Although the results provide support for the psychometric strengths of the Brazilian version of the MMQ, additional studies are needed to further examine its psychometric properties, particularly in a larger sample. 

## APPENDIX

Questionário Multifatorial de Memória (MMQ)(Simon et al., 2016)

MMQ-Sentimento Como eu me sinto sobre a minha memória

Folha de Aplicação

Abaixo estão afirmações sobre sentimentos que as pessoas talvez tenham sobre a própria memória. Leia cada frase e decida se você concorda.

**Table:** Pense em como você tem se sentido nas ***últimas duas semanas.*** Em seguida, marque a coluna apropriada.

		Concordo totalmente	Concordo	Indeciso	Disorcordo	Discordo totalmente
1	Geralmente eu estou contente com a minha memória.					
2	Existe algo seriamente errado com a minha memória.					
3	Se algo é importante eu provavelmente irei lembrar.					
4	Quando eu esqueço algo, eu tenho medo de ter um sério problema de memória, como a doença de Alzheimer.					
5	A minha memória é pior do que a memória de outras pessoas da minha idade.					
6	Eu tenho confiança na minha capacidade de lembrar informações.					
7	Eu me sinto infeliz quando penso na minha memória.					
8	Eu me preocupo que os outros notem que a minha memória não está tão boa.					
9	Quando eu tenho dificuldade para lembrar algo, eu não sou tão severo / exigente comigo mesmo (a).					
10	Eu estou preocupado (a) com a minha memória.					
11	Minha memória está piorando muito ultimamente.					
12	Geralmente eu estou satisfeito (a) com a minha memória.					
13	Eu não me aborreço quando eu tenho problemas para lembrar algo.					
14	Eu fico preocupado (a) que eu possa esquecer algo importante.					
15	Eu estou envergonhado (a) com a minha memória.					
16	Eu fico irritado (a) ou chateado (a) comigo mesmo (a) quando eu esqueço algo.					
17	A minha memória é boa para a minha idade.					
18	Eu me preocupo com a minha memória.					

## APPENDIX

Questionário Multifatorial de Memória (MMQ) (Simon et al., 2016)

MMQ-Habilidade

Falhas de Memória

Folha de Aplicação

**Table:** Abaixo há uma lista de falhas de memória comuns que as pessoas cometem. Decida com que frequência que você tem cometido cada uma destas nas ***últimas duas semanas.*** em seguida, marque a coluna apropriada.

		O tempo todo	Frequentemente	Às vezes	Raramente	Nunca
1	Esquece de pagar uma conta a tempo.					
2	Guarda no lugar errado algo que usa diariamente, como chaves ou óculos.					
3	Tem dificuldade em lembrar um número de telefone que você acabou de olhar.					
4	Não se lembra do nome de alguém que você acabou de conhecer.					
5	Esquece algo você queria trazer com você.					
6	Esquece um compromisso.					
7	Esquece o que você estava indo fazer, por exemplo, entra em um quarto e esquece o que você foi fazer lá.					
8	Esquece de cumprir um dever.					
9	Em uma conversa, tem dificuldade para encontrar uma determinada palavra que quer.					
10	Tem dificuldade em lembrar detalhes de um texto de revista ou jornal que leu no começo do dia.					
11	Esquece de tomar remédios.					
12	Não se lembra do nome de alguém que você conhece há algum tempo.					
13	Esquece de dar um recado.					
14	Esquece o que você ia dizer numa conversa.					
15	Esquece aniversários ou datas comemorativas que você costumava saber bem.					
16	Esquece números de telefone que você usa com frequência.					
17	Conta a mesma história ou a mesma piada porque esqueceu que já tinha contado.					
18	Confunde o lugar de algo que guardou há alguns dias atrás.					
19	Esquece de comprar algo que você pretendia comprar.					
20	Esquece os detalhes de uma conversa recente.					

## APPENDIX

Questionário Multifatorial de Memória (MMQ) (Simon et al., 2016)

MMQ-Estratégia

Estratégias de Memória

Folha de Aplicação

As pessoas frequentemente usam diferentes auxílios ou estratégias para lembrar as coisas. Diversas estratégias estão listadas abaixo.

**Table:** Decida com que frequência você tem usado cada uma delas nas ***últimas duas semanas.*** Em seguida marque a coluna apropriada.

		O tempo todo	Frequentemente	Às vezes	Raramente	Nunca
1	Usa um alarme ou despertador para lembrar de fazer alguma coisa.					
2	Pede que alguém o ajude a lembrar de algo ou lembre que você precisa fazer algo.					
3	Cria uma rima com a informação que você quer lembrar.					
4	Na sua mente, cria uma imagem visual de algo que você queira se lembrar, como associar um nome a um rosto.					
5	Anota coisas num calendário, tais como compromissos ou coisas que precisam ser feitas.					
6	Segue o alfabeto, letra por letra, para ver se você lembra de um nome ou uma palavra.					
7	Organiza as informações que você precisa lembrar, como por exemplo, organizar a sua lista de supermercado de acordo com grupos de comidas.					
8	Fala algo em voz alta com o objetivo de se lembrar, como um número de telefone que você acabou de olhar.					
9	Usa uma rotina para se lembrar de coisas importantes, como checar se você está levando a sua carteira e a sua chave quando sai de casa.					
10	Faz uma lista, como uma lista de supermercado ou de coisas a serem feitas.					
11	Elabora mentalmente algo que você quer se lembrar, como por exemplo, prestar atenção em detalhes.					
12	Coloca algo em um lugar que chame a sua atenção para lembrá-lo (a) de fazer algo, como colocar o guarda chuva na frente da porta para você se lembrar de levá-lo com você.					
13	Repete algo para você mesmo (a) em intervalos cada vez maiores, assim você irá se lembrar.					
14	Cria uma estória para juntar informações que você quer se lembrar.					
15	Escreve em uma agenda coisas que você quer se lembrar.					
16	Cria uma sigla com as primeiras letras em uma lista de coisas que você quer lembrar, tais como limão, uva e amendoim (LUA).					
17	Intencionalmente se concentra muito em algo para poder se lembrar.					
18	Escreve um bilhete ou um lembrete para você mesmo (diferente de calendário ou agenda).					
19	Refaz mentalmente as etapas de algo que você fez com o intenção de lembrar algo, como o lugar de alguma coisa que você não lembra onde está. coluna apropriada.					

## APPENDIX

Questionário Multifatorial de Memória (MMQ) (Simon et al., 2016)

MMQ-Sentimento Como eu me sinto sobre a minha memória

Folha de Correção

Abaixo estão afirmações sobre sentimentos que as pessoas talvez tenham sobre a própria memória. Leia cada frase e decida se você concorda.

**Table:** Pense em como você tem se sentido nas ***últimas duas semanas.*** Em seguida, marque a coluna apropriada.

		Concordo totalmente	Concordo	Indeciso	Disorcordo	Discordo totalmente
1	Geralmente eu estou contente com a minha memória.	4	3	2	1	0
2	Existe algo seriamente errado com a minha memória.	0	1	2	3	4
3	Se algo é importante eu provavelmente irei lembrar.	4	3	2	1	0
4	Quando eu esqueço algo, eu tenho medo de ter um sério problema de memória, como a doença de Alzheimer.	0	1	2	3	4
5	A minha memória é pior do que a memória de outras pessoas da minha idade.	0	1	2	3	4
6	Eu tenho confiança na minha capacidade de lembrar informações.	4	3	2	1	0
7	Eu me sinto infeliz quando penso na minha memória.	0	1	2	3	4
8	Eu me preocupo que os outros notem que a minha memória não está tão boa.	0	1	2	3	4
9	Quando eu tenho dificuldade para lembrar algo, eu não sou tão severo / exigente comigo mesmo (a).	4	3	2	1	0
10	Eu estou preocupado (a) com a minha memória.	0	1	2	3	4
11	Minha memória está piorando muito ultimamente.	0	1	2	3	4
12	Geralmente eu estou satisfeito (a) com a minha memória.	4	3	2	1	0
13	Eu não me aborreço quando eu tenho problemas para lembrar algo.	4	3	2	1	0
14	Eu fico preocupado (a) que eu possa esquecer algo importante.	0	1	2	3	4
15	Eu estou envergonhado (a) com a minha memória.	0	1	2	3	4
16	Eu fico irritado (a) ou chateado (a) comigo mesmo (a) quando eu esqueço algo.	0	1	2	3	4
17	A minha memória é boa para a minha idade.	4	3	2	1	0
18	Eu me preocupo com a minha memória.	0	1	2	3	4

## APPENDIX

Questionário Multifatorial de Memória (MMQ) (Simon et al., 2016)

MMQ-Habilidade

Falhas de Memória

Folha de Correção

**Table:** Abaixo há uma lista de falhas de memória comuns que as pessoas cometem. Decida com que frequência que você tem cometido cada uma destas nas ***últimas duas semanas.*** em seguida, marque a coluna apropriada.

		O tempo todo	Frequentemente	Às vezes	Raramente	Nunca
1	Esquece de pagar uma conta a tempo.	0	1	2	3	4
2	Guarda no lugar errado algo que usa diariamente, como chaves ou óculos.	0	1	2	3	4
3	Tem dificuldade em lembrar um número de telefone que você acabou de olhar.	0	1	2	3	4
4	Não se lembra do nome de alguém que você acabou de conhecer.	0	1	2	3	4
5	Esquece algo você queria trazer com você.	0	1	2	3	4
6	Esquece um compromisso.	0	1	2	3	4
7	Esquece o que você estava indo fazer, por exemplo, entra em um quarto e esquece o que você foi fazer lá.	0	1	2	3	4
8	Esquece de cumprir um dever.	0	1	2	3	4
9	Em uma conversa, tem dificuldade para encontrar uma determinada palavra que quer.	0	1	2	3	4
10	Tem dificuldade em lembrar detalhes de um texto de revista ou jornal que leu no começo do dia.	0	1	2	3	4
11	Esquece de tomar remédios.	0	1	2	3	4
12	Não se lembra do nome de alguém que você conhece há algum tempo.	0	1	2	3	4
13	Esquece de dar um recado.	0	1	2	3	4
14	Esquece o que você ia dizer numa conversa.	0	1	2	3	4
15	Esquece aniversários ou datas comemorativas que você costumava saber bem.	0	1	2	3	4
16	Esquece números de telefone que você usa com frequência.	0	1	2	3	4
17	Conta a mesma história ou a mesma piada porque esqueceu que já tinha contado.	0	1	2	3	4
18	Confunde o lugar de algo que guardou há alguns dias atrás.	0	1	2	3	4
19	Esquece de comprar algo que você pretendia comprar.	0	1	2	3	4
20	Esquece os detalhes de uma conversa recente.	0	1	2	3	4

## APPENDIX

Questionário Multifatorial de Memória (MMQ) (Simon et al., 2016)

MMQ-Estratégia

Estratégias de Memória

Folha de Correção

As pessoas frequentemente usam diferentes auxílios ou estratégias para lembrar as coisas. Diversas estratégias estão listadas abaixo.

**Table:** Decida com que frequência você tem usado cada uma delas nas ***últimas duas semanas.*** Em seguida marque a coluna apropriada.

		O tempo todo	Frequentemente	Às vezes	Raramente	Nunca
1	Usa um alarme ou despertador para lembrar de fazer alguma coisa.	4	3	2	1	0
2	Pede que alguém o ajude a lembrar de algo ou lembre que você precisa fazer algo.	4	3	2	1	0
3	Cria uma rima com a informação que você quer lembrar.	4	3	2	1	0
4	Na sua mente, cria uma imagem visual de algo que você queira se lembrar, como associar um nome a um rosto.	4	3	2	1	0
5	Anota coisas num calendário, tais como compromissos ou coisas que precisam ser feitas.	4	3	2	1	0
6	Segue o alfabeto, letra por letra, para ver se você lembra de um nome ou uma palavra.	4	3	2	1	0
7	Organiza as informações que você precisa lembrar, como por exemplo, organizar a sua lista de supermercado de acordo com grupos de comidas.	4	3	2	1	0
8	Fala algo em voz alta com o objetivo de se lembrar, como um número de telefone que você acabou de olhar.	4	3	2	1	0
9	Usa uma rotina para se lembrar de coisas importantes, como checar se você está levando a sua carteira e a sua chave quando sai de casa.	4	3	2	1	0
10	Faz uma lista, como uma lista de supermercado ou de coisas a serem feitas.	4	3	2	1	0
11	Elabora mentalmente algo que você quer se lembrar, como por exemplo, prestar atenção em detalhes.	4	3	2	1	0
12	Coloca algo em um lugar que chame a sua atenção para lembrá-lo (a) de fazer algo, como colocar o guarda chuva na frente da porta para você se lembrar de levá-lo com você.	4	3	2	1	0
13	Repete algo para você mesmo (a) em intervalos cada vez maiores, assim você irá se lembrar.	4	3	2	1	0
14	Cria uma estória para juntar informações que você quer se lembrar.	4	3	2	1	0
15	Escreve em uma agenda coisas que você quer se lembrar.	4	3	2	1	0
16	Cria uma sigla com as primeiras letras em uma lista de coisas que você quer lembrar, tais como limão, uva e amendoim (LUA).	4	3	2	1	0
17	Intencionalmente se concentra muito em algo para poder se lembrar.	4	3	2	1	0
18	Escreve um bilhete ou um lembrete para você mesmo (diferente de calendário ou agenda).	4	3	2	1	0
19	Refaz mentalmente as etapas de algo que você fez com o intenção de lembrar algo, como o lugar de alguma coisa que você não lembra onde está. coluna apropriada.	4	3	2	1	0
